# A Self-Calibrating Localization Solution for Sport Applications with UWB Technology

**DOI:** 10.3390/s22239363

**Published:** 2022-12-01

**Authors:** Marco Piavanini, Luca Barbieri, Mattia Brambilla, Mattia Cerutti, Simone Ercoli, Andrea Agili, Monica Nicoli

**Affiliations:** 1 Dipartimento di Elettronica, Informazione e Bioingegneria, Politecnico di Milano, Via Ponzio 34/5, 20133 Milan, Italy; 2 Department of Management, Economics and Industrial Engineering, Politecnico di Milano, Via Lambruschini 4/B, 20156 Milan, Italy; 3 Tracking4Fun S.r.l., Via delle Panche 140, 50141 Florence, Italy

**Keywords:** UWB, localization, antenna delay, anchor self-localization, outlier detection, Isolation forest, Gauss–Newton algorithm, Levenberg–Marquardt algorithm

## Abstract

This study addressed the problem of localization in an ultrawide-band (UWB) network, where the positions of both the access points and the tags needed to be estimated. We considered a fully wireless UWB localization system, comprising both software and hardware, featuring easy plug-and-play usability for the consumer, primarily targeting sport and leisure applications. Anchor self-localization was addressed by two-way ranging, also embedding a Gauss–Newton algorithm for the estimation and compensation of antenna delays, and a modified isolation forest algorithm working with low-dimensional set of measurements for outlier identification and removal. This approach avoids time-consuming calibration procedures, and it enables accurate tag localization by the multilateration of time difference of arrival measurements. For the assessment of performance and the comparison of different algorithms, we considered an experimental campaign with data gathered by a proprietary UWB localization system.

## 1. Introduction

Owing to the integration of low power networking, microsensing, and data analytics tools (e.g., cloud based), the Internet of Things (IoT) is nowadays becoming a reality in everyday applications, such as smart industries [1], healthcare [2,3], and smart homes [4]. An emerging field of application is related to sport and leisure activities owing to the increasing availability of smart wearables [5], where sensors’ data collection and analysis allow the monitoring of biological and kinematic parameters of athletes, developing smart solutions for injuries prevention, activity recognition and tactics. Therein, accurate localization [6,7] is a critical requirement, and the commonly used Global Navigation Satellite Systems (GNSSs) are no longer enough or may even be unavailable, such as in indoor environments. In sports applications, localization with submeter accuracy is mandatory as multiple players fit within a restricted area and are often close each other. For example, most game sports typically require accurate positioning, in the order of 15–20 cm, while also demanding frequent location updates (e.g., 10–15 Hz) [8]. Furthermore, seamless solutions should be developed so as to provide reliable position estimates in outdoor, indoor, or mixed scenarios. Such stringent requirements call for novel signal-processing tools and/or specific positioning technologies. A promising possibility is represented by the employment of dedicated Wireless Sensor Networks (WSNs), where a set of sensor nodes equipped with wireless devices is able to detect signals and communicate with each other [9,10]. WSNs exploit radio signals to extract location-dependent measurements that characterize the distance and/or the angle, typically, among any two connected devices. Depending on the required localization accuracy of the considered application, several technologies and methodologies can be used [11]. Among all the available solutions, Ultrawide-Band (UWB) technology [12,13,14,15] is one of the most promising. UWB positioning networks provide accurate ranging, reaching decimeter-level accuracy thanks to the large bandwidth [16,17]. Moreover, UWB signals easily penetrate through many materials [18] and have a very short duration of pulses that makes them more robust against interference and less sensitive to multipath propagation effects [19,20,21].

In UWB localization, a network of connected nodes, called anchors or Access Points (APs), is used to monitor an area of interest and localize users equipped with tags. In fixed deployments, the exact position of each AP is calculated within a preliminary calibration phase and then used as an input parameter by a localization algorithm. In temporary deployments, instead, a Self-Localization (SL) procedure to estimate the APs position is required, where each anchor node exchanges ranging messages with the neighbors to reconstruct the network geometry. Then, the tag localization can take place, where all anchors collect measurements for estimating the tag position, such as Time of Arrival (TOA), Angle of Arrival (AOA), Time Difference of Arrival (TDOA), and Received Signal Strength (RSS) [22,23,24,25]. In this study, we focused on a UWB localization system for sport applications, where SL is performed with periodic exchange of TOA measurements, and the tags are localized via multilateration over TDOA measurements.

### 1.1. Related Works

The available SL approaches use ranging [26], angle [27], or inertial measurements [28]. Fusion algorithms are required to integrate such measurement  [29], like multidimensional scaling [30], semidefinite programming [31], or trilateration approaches [32,33].

The anchors’ SL is affected by the errors generated by the electronic components of UWB devices as well as of non-ideal propagation conditions. In particular, delays are introduced by the receiving and/or transmitting antennas, called antenna delays. Compensation procedures have been devised to correct such delays. Examples are Particle Swarm Optimization (PSO) [34] and nonparametric method taking into account the antenna delay statistics [35]. Other procedures rely on genetic-like algorithms [36] or customized ranging message schemes [37]. The aforementioned compensation methods target the estimation of the overall antenna delay among any pair of UWB nodes. Nevertheless, isolating the specific transmitting and/or receiving contribution on the antenna delay of UWB nodes is required when dealing with one-way measurement procedures (such as TDOA), as the same UWB nodes are used for SL and tag localization. Algebraic solutions were proposed in [38], while a semidefinite programming framework was devised in [39]. Other methods rely on supervised learning techniques [40] that aim to learn them with a data-driven approach.

UWB systems can also be affected by outliers, due to inaccuracies in ranging estimation, synchronization issues, and packet decoding errors. Outlier identification and removal are other crucial steps for obtaining precise ranging measurements. Supervised learning techniques relying on recurrent neural networks and support vector machines were proposed by [41,42], respectively. Unsupervised methods have also been developed, focusing on k-nearest neighbor [43], local outlier factor [44], and Isolation Forest Algorithm (IFA)  [45] approaches. Maximum likelihood estimation was considered in [46] to filter out outliers and improve the localization accuracy.

Concerning tag localization, Least Square (LS) solutions [47] have been successfully applied for position estimation exploiting Gauss–Newton (GN) or Levenberg–Marquardt (LM) algorithms. Other popular solutions for localization are RSS-based fingerprinting techniques [48,49]. Lastly, Bayesian approaches are also common [15], as they integrate side information such as environment conditions [50], map information [51,52], sensor fusion [53,54], or channel impulse response [55].

### 1.2. Study Contributions

We designed a fully wireless (i.e., not requiring any cabling) UWB localization system that relies on Decawave DW1000 devices and integrates augmentation algorithms to improve positioning accuracy. The new platform (both software and hardware) provides a friendly interface and installation is easy (i.e., without tedious calibration procedures) such that non-expert and non-trained users can easily deploy this system anytime and anywhere. These features allow the system to be positioned for sports markets as a main target. The proposed system was designed accounting for the main sources of errors degrading the localization accuracy and integrating novel countermeasures as detailed below:

- Time of Flight (TOF) measurements among APs are used to reconstruct the network geometry in the SL procedure. An algorithm based on GN is used to handle the errors due to antenna delays in the TOF estimate. The algorithm relies on an iterative procedure for estimating the antenna delay by minimizing the difference between the true and estimated TOF at each UWB node. Compared with other methods such as [34], the proposed method converges to the optimal solution and can be applied to conventional ranging schemes, avoiding the definition of custom messages as in [37].
- Outlier identification and removal is here addressed by proposing a modified IFA. Compared with the standard IFA [45], the proposed algorithm can tackle a very-low-dimensional set of measurements while achieving high accuracy in detecting outliers. This method was designed due to the low number of TOF measurements available for anchors’ SL.
- For anchors’ SL, we developed an iterative algorithm that exploits the filtered TOF measurements to reconstruct the system geometry. The algorithm, inspired by [26], iteratively searches for an AP configuration that minimizes the residual error between the TOF measurements provided by the UWB system and the distances extracted from the positioned anchors. Rather than relying on complex optimization procedures, such as the ones proposed in [31], the developed algorithm relies on GN algorithm which is computationally efficient.
- Tag localization is achieved by multilateration of TDOA measurements. To compensate for the TDOA antenna delay at the anchors, we statistically modeled the TDOA measurements and extracted the corresponding delay through an inversion operation. Compared with the other approaches available in the literature, the proposed approach does not require any training procedure, as opposed to [40] or complex optimization procedures as in [39].

An experimental campaign was conducted at the IoTLab facility of the Politecnico di Milano to extract raw UWB data and assess all the developed techniques. The aim of the analysis was to evaluate the benefits of using algorithms for outlier identification as well as for antenna delay calibration. The experimental results indicated that the developed techniques are particularly effective in enhancing the positioning accuracy of the anchors, especially when the antenna delay and the outliers are properly handled. Then, we evaluated the tag localization performance considering the GN and LM algorithms. The numerical results showed that when combining all the proposed compensation procedures, the accuracy in tag positioning using the anchors’ SL algorithm is comparable to that when knowing the true positions of the AP in advance.

Part of this study was presented in [56], where the problem of Asymmetric Double-Sided Two-Way Ranging (ADS-TWR) antenna delay calibration and its impact on the anchor SL were studied. Here, we further detail the calibration for SL, also including outlier identification and removal. Moreover, we assessed the impact of accurate SL on tag SL, analyzing both the position error and the availability of the estimate.

The remainder of this paper is structured as follow: Section 2 outlines the ranging methods; Section 3 describes the impact of antenna delay and compensation methodologies. Section 4 describes the modified IFA for outlier detection; Section 5 describes the anchors’ SL and tag localization LS methods. In Section 6, we discuss the experimental setup and define the performance metrics. Section 7 presents the results of the experimental campaign; lastly, Section 8 discusses the conclusions and objectives for future work.

## 2. System and Measurement Models

We considered an UWB network with *N* static APs, placed at the same height and at positions $p_{i}={\left[p_{i,x}\;\;\;p_{i,y}\right]}^{T}\in R^{2},\forall i\in \left\{1,\;\cdots \;,N\right\},$ and monitoring a three-dimensional (3D) area $U\in R^{3}$. The APs had to localize a set of UWB user tags by multilateration of TDOA measurements. An SL procedure was carried out during the installation phase to allow the APs to estimate their positions by the exchange of ranging messages. The measurements used for anchors’ SL are discussed in Section 2.1, followed by details on the measurement model for tag localization in Section 2.2.

### 2.1. Measurements for AP Network Localization

To estimate the positions of APs (i.e., for network SL), we considered the ADS-TWR algorithm [57] as it offers more resilient capabilities for synchronization issues compared with one-way ranging procedures [58]. It uses TOF estimates between anchor pairs to retrieve the distance by the exchange of ad hoc ranging messages. Furthermore, compared with symmetric double-sided schemes, ADS-TWR reduces the overall number of messages exchanged between anchor pairs, limiting the impact of clock offsets [58].

**Figure 1:**
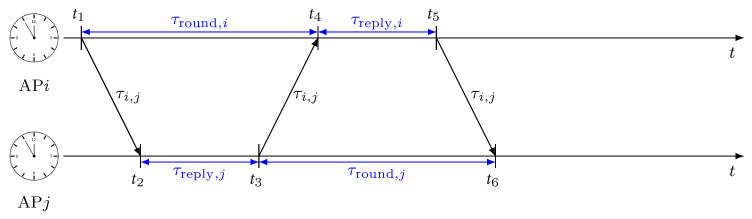
Message exchange with ADS-TWR procedure.

ADS-TWR relies on the exchange of three messages between two APs for TOF estimation, as depicted in Figure 1. Let us suppose the pair (AP*i*, AP*j*) of anchors, with i≠j, involved in the ADS-TWR message exchange. At time $t_{1}$, AP*i* sends a ranging message to AP*j*, which receives it at time $t_{2}$ and replies back at time $t_{3}$, after waiting for a fixed time $\tau_{\mathrm{reply},j}$. Once AP*i* receives the message from AP*j* at time $t_{4}$, it waits for a fixed time $\tau_{\mathrm{reply},i}$ and then sends another message at time $t_{5}$. The procedure ends up with the reception of the message by AP*j* at time $t_{6}$. The goal of the ADS-TWR procedure is to estimate the TOF $\tau_{ij}$ between the two APs, which amounts to [59]

(1) $$\begin{array}{c} {\hat{\tau }}_{ij}=\frac{\tau_{\mathrm{round},i}\;\tau_{\mathrm{round},j}-\tau_{\mathrm{reply},i}\;\tau_{\mathrm{reply},j}}{\tau_{\mathrm{reply},i}+\tau_{\mathrm{reply},j}+\tau_{\mathrm{round},i}+\tau_{\mathrm{round},j}}\;, \end{array}$$

where $\tau_{\mathrm{round},i}=t_{4}-t_{1}=2\tau_{ij}+\tau_{\mathrm{reply},j}$ and $\tau_{\mathrm{reply},i}$ are the Round Trip Time (RTT) and reply time of anchor *i*, respectively. Similarly, $\tau_{\mathrm{round},j}=t_{6}-t_{3}=2\tau_{ij}+\tau_{\mathrm{reply},i}$ and $\tau_{\mathrm{reply},j}$ are the RTT and reply time of anchor *j*, respectively. From the TOF, the estimated distance between a pair of anchors is ${\hat{d}}_{ij}=c\;{\hat{\tau }}_{ij}$, where *c* is the speed of light.

**Figure 2:**
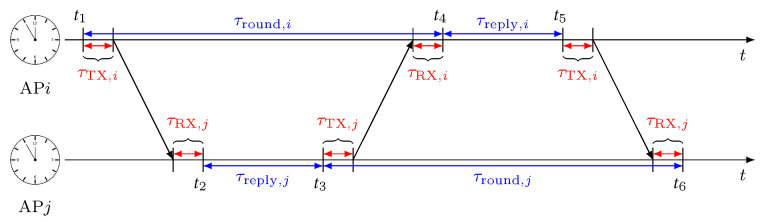
Message exchange with ADS-TWR procedure in presence of antenna delays.

The TOF in (1) does not take into account the additional errors that may be introduced during range estimation. The radio signals used to exchange ranging messages are subject to the undesired delays introduced by the radio transceivers. These delays, referred to as antenna delays [34], need to be properly characterized and estimated to minimize their degradation impact. The impact of antenna delays on the ADS-TWR message scheme is depicted in Figure 2, where we highlight the contributions of the antenna delay at anchor *i*, i.e., $\tau_{\mathrm{TX},i}$ and $\tau_{\mathrm{RX},i}$, and at anchor *j*, i.e., $\tau_{\mathrm{TX},j}$ and $\tau_{\mathrm{RX},j}$. Note that we decomposed the antenna delay into transmitter (TX) and receiver (RX) contributions as physical antenna implementations may be subject to manufacturing imperfections leading to different antenna delays at TX and RX. The RTT introduced in (1) was reformulated to take into account these delays as

(2) $$\begin{array}{cc} \tau_{\mathrm{round},i}= & t_{4}-t_{1}=2\tau_{ij}+\tau_{reply,j}+\tau_{AD,ij} \end{array}$$

(3) $$\begin{array}{cc} \tau_{\mathrm{round},j}= & t_{6}-t_{3}=2\tau_{ij}+\tau_{reply,i}+\tau_{AD,ij} \end{array}$$

with

(4) $$\tau_{AD,ij}=\underset{\tau_{D,i}}{\underbrace{\tau_{\mathrm{TX},i}+\tau_{\mathrm{RX},i}}}+\underset{\tau_{D,j}}{\underbrace{\tau_{\mathrm{TX},j}+\tau_{\mathrm{RX},j}}}\;,$$

where $\tau_{\mathrm{AD},ij}$ is the total antenna delay introduced by the anchor pair *i* and *j*; $\tau_{D,i}$ and $\tau_{D,j}$ are the individual antenna delay contributions of anchors *i* and *j*, respectively. If we do not take into account effect of the antenna delays in (1), the TOF estimation would be

(5) $$\Delta \tau_{ij}={\hat{\tau }}_{ij}-\tau_{ij}=\frac{\tau_{AD,ij}}{2}\;.$$

By accounting for the antenna delays, the estimated TOF in (1) transforms into the following estimate:

(6) $${\hat{\tau }}_{ij}\left(\tau_{\mathrm{AD},ij}\right)=\frac{\tau_{\mathrm{round},i}\tau_{\mathrm{round},j}-\tau_{\mathrm{reply},i}\tau_{\mathrm{reply},j}-\left(\tau_{\mathrm{reply},i}+\tau_{\mathrm{reply},j}\right)\tau_{\mathrm{AD},ij}-\tau_{\mathrm{AD},ij}^{2}}{\tau_{\mathrm{reply},i}+\tau_{\mathrm{reply},i}+\tau_{\mathrm{round},i}+\tau_{\mathrm{round},j}+2\tau_{\mathrm{AD},ij}}\;.$$

Then, the distance between the two APs is ${\hat{d}}_{ij}=c\;{\hat{\tau }}_{ij}\left(\tau_{\mathrm{AD},ij}\right)$. By knowing the antenna delays and using (6), it is possible to eliminate the positive bias in the TOF estimate introduced by the UWB modules.

### 2.2. Measurements for Tag Localization

**Figure 3:**
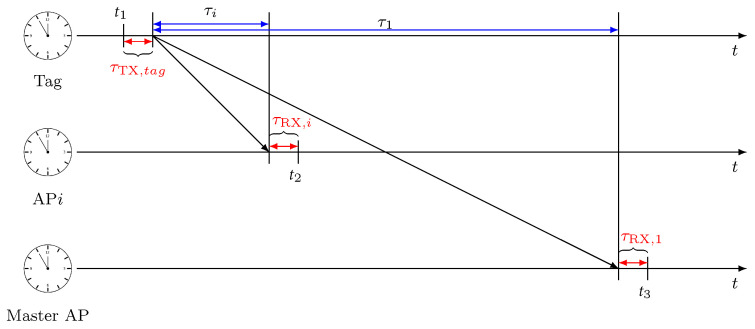
TDOA estimation procedure between AP *i* and master AP in presence of antenna delays.

TDOAs are estimated as the difference between pseudo ranges gathered at the reference station (i.e., the master anchor AP1) and any other AP*i*, for i=2,⋯,N. The procedure for estimating the TDOA among anchor pair {1,i} is depicted in Figure 3, considering the contribution of the antenna delays. At time $t_{1}$, the tag sends a message that carries the transmission antenna delay $\tau_{\mathrm{TX},tag}$. This message is received by both AP*i* and AP1 at times $t_{2}$ and $t_{3}$, respectively. After the successful reception of the message, AP*i* can extract the TOF as

(7) $${\hat{\tau }}_{i}=t_{2}-t_{1}=\tau_{i}+\tau_{\mathrm{TX},tag}+\tau_{\mathrm{RX},i}\;,$$

where $\tau_{\mathrm{TX},tag}$ is the transmitting antenna delay of the tag, and $\tau_{\mathrm{RX},i}$ is the receiving antenna delay of AP*i*. Similarly, the master obtains the TOF as

(8) $${\hat{\tau }}_{1}=t_{3}-t_{1}=\tau_{1}+\tau_{\mathrm{TX},tag}+\tau_{\mathrm{RX},1}\;.$$

At this point, the TDOA measurement between the AP pair {1,i} can be computed as

(9) $$\rho_{i,1}=c\;\left({\hat{\tau }}_{i}-{\hat{\tau }}_{1}\right)=c\;\left(\tau_{i}+\tau_{\mathrm{TX},tag}+\tau_{\mathrm{RX},i}-\tau_{1}-\tau_{\mathrm{TX},tag}-\tau_{\mathrm{RX},1}\right)=d_{i}-d_{1}+b_{i}-b_{1}\;,$$

where $d_{i}=c\;\tau_{i}$ and $d_{1}=c\;\tau_{1}$ are the true ranges between the *i*th AP and the tag and between the master and the tag, respectively. It should be noted that the TDOA error originates only from the receiving antenna delay of the anchors, as the transmitting antenna delay of the tag is eliminated. The overall set of TDOA measurements $\rho ={\left[\rho_{2,1}\;\cdots \rho_{N,1}\right]}^{T}$ can be expressed in compact notation as

(10) ρ=h(u)+Δb,

where $u={\left[u_{x}\;u_{y}\;u_{z}\right]}^{T}$ is the 3D tag position, $h\left(u\right)={\left[h_{2,1}\left(u\right)\cdots h_{N,1}\left(u\right)\right]}^{T}$ with $h_{i,1}\left(u\right)=d_{i}-d_{1}$ being the measurement model, and $\Delta b={\left[\Delta b_{2}\cdots \Delta b_{N}\right]}^{T}$ with $\Delta b_{i}=b_{i}-b_{1}$ and $b_{i}=c\tau_{RX,i}$ the antenna delay’s bias vector.

## 3. Antenna Delay Calibration

In this section, we detail the proposed methodology to compensate for the antenna delays described in Section 2. Specifically, we address the problem of Antenna Delay Calibration (ADC) of ADS-TWR in Section 3.1, whereas Section 3.2 focuses on the calibration of the TDOA antenna delay.

### 3.1. ADS-TWR Antenna Delay Calibration

Rather than relying on PSO, we use a GN algorithm for AD compensation, which is more computationally efficient and provides optimal solutions, as it does not randomly search in the solution space. The goal of ADC is to estimate the total antenna delay introduced by each AP*i*, ∀i∈{1,⋯,N}. By combining all the total antenna delays into the vector $\tau_{AD}={\left[\tau_{AD,12}\cdots \tau_{AD,1N}\;\tau_{AD,21}\cdots \tau_{AD,(N-1)N}\right]}^{T}$ and  the total individual antenna delays into $\tau_{D}={\left[\tau_{D,1}\cdots \tau_{D,N}\right]}^{T}$, it is possible to rewrite (4) as

(11) $$\tau_{AD}=M\tau_{D}\;,$$

where $M=\left[m_{ij}\right]$ with $m_{ij}\in \left\{0,1\right\}$ is a transformation matrix encoding the relations $\tau_{AD,ij}=\tau_{D,i}+\tau_{D,j}$. Then, the antenna delays of each anchor are estimated according to

(12) $$\tau_{D}={\left(M^{T}M\right)}^{-1}M\tau_{AD}\;.$$

Solving (12) requires the knowledge of $\tau_{AD}$, whose elements can be extracted using GN methods as detailed in the following.

The ADC process considers a generic deployment of *N* APs in a given area, in which the ADS-TWR procedure is used to measure the TOF in (6) and to estimate the distances between all the anchor pairs. It consists of exchanging multiple successive ranging messages carrying TOF information. Let ${\hat{\tau }}_{ij}^{\left(m\right)}$ denote a single TOF estimate between AP*i* and AP*j*, with ${\hat{d}}_{ij}^{\left(m\right)}=c\;{\hat{\tau }}_{ij}^{\left(m\right)}$, the associated range; and with $D_{ij}={\left\{{\hat{d}}_{ij}^{\left(m\right)}\right\}}_{m=1}^{M_{ij}}$, the whole set of $M_{ij}$ distance measurements collected at AP pair {i,j}. The total contribution of the antenna delay for AP*i* and AP*j*, i.e., $\tau_{AD,ij}$, is obtained solving the following optimization problem:

(13) $${\hat{\tau }}_{AD,ij}=\arg\min_{\tau_{\mathrm{AD},ij}}\sum_{m=1}^{M_{ij}}{\left(d_{ij}-{\hat{d}}_{ij}^{\left(m\right)}\left(\tau_{\mathrm{AD},ij}\right)\right)}^{2}=\arg\min_{\tau_{\mathrm{AD},ij}}\sum_{m=1}^{M_{ij}}c^{2}{\left(\tau_{ij}-{\hat{\tau }}_{ij}^{\left(m\right)}\left(\tau_{\mathrm{AD},ij}\right)\right)}^{2}.$$

Let us suppose a solution ${\bar{\tau }}_{AD,ij}$ for (13) is available from a previous time step. The updated estimate is computed according to the LS criterion as

(14) $${\hat{\tau }}_{AD,ij}\leftarrow {\bar{\tau }}_{AD,ij}+\frac{h{\left({\bar{\tau }}_{AD,ij}\right)}^{T}\Delta \tau }{∥h\left({\bar{\tau }}_{AD,ij}\right){∥}^{2}}\;,$$

where $h\left(\tau_{AD,ij}\right)={\left[\partial {\hat{\tau }}_{ij}^{\left(1\right)}/\partial \tau_{AD,ij}\cdots \partial {\hat{\tau }}_{ij}^{\left(M_{ij}\right)}/\partial \tau_{AD,ij}\right]}^{T}$ and $\Delta \tau ={\left[\tau_{ij}-{\hat{\tau }}_{ij}^{\left(1\right)}\cdots \tau_{ij}-{\hat{\tau }}_{ij}^{\left(M_{ij}\right)}\right]}^{T}$. The algorithm starts from a random guess at time t=0, and then, at each iteration, generates a new solution using (14): it stops after reaching a maximum number of iterations or when the residual $|\Delta \tau_{AD,ij}\left|=\right|{\hat{\tau }}_{AD,ij}-{\bar{\tau }}_{AD,ij}|$ becomes lower than a certain threshold. This procedure is iterated over all possible anchor pairs to populate the vector $\tau_{AD}$. Thus, from $\tau_{AD}$, it is possible to estimate $\tau_{D}$ using (12). The entries of $\tau_{D}$ are then saved in the corresponding UWB module memory of the anchors. Every time AP*i* and AP*j* perform an ADS-TWR procedure, AP*i* includes $\tau_{D,i}$ into the payload of the first message. Upon completion of the message scheme, AP*j* reconstructs $\tau_{AD,ij}$ from its antenna delay $\tau_{D,j}$ and the one received from AP*i*, i.e., $\tau_{D,i}$, using (4) to calculate the TOF according to (6).

### 3.2. TDOA Antenna Delay Calibration

To use more accurate TDOA measurement for tag localization, the antenna delay must be compensated, as discussed in Section 2.2. The compensation procedure requires to estimate only the receiving antenna delay contribution at the AP as the antenna delay of the tag is eliminated when the TDOA is computed. The algorithm for ADS-TWR proposed in Section 3.1 is able to estimate the total antenna delay $\tau_{D,i}$ introduced by each AP, but it cannot isolate the TX and RX contributions, i.e., $\tau_{\mathrm{TX},i}$ and $\tau_{\mathrm{RX},i}$. To overcome this limitation, we designed a novel procedure to extract the RX contribution $\tau_{\mathrm{RX},i}$ and compensate for the TDOA measurement. Suppose that $N_{t}$ TDOA measurements ${\left\{\rho_{t}\right\}}_{t=1}^{N_{k}}$ are estimated using tag with known positions ${\left\{u_{t}\right\}}_{t=1}^{N_{t}}$. We can use the $N_{t}$ TDOA measurements to calculate the vector Δb using the sample mean estimator as

(15) $$\Delta b=\frac{1}{N_{t}}\sum_{t=1}^{N_{t}}\left(\rho_{t}-h\left(u_{t}\right)\right)\;.$$

We can then write the vector Δb as a linear system

(16) Δb=Fb,

where the elements of matrix $F\in R^{(N-1)xN}$ are constructed as

(17) $$F_{ij}=\left\{\begin{array}{c} -1\;j=1\wedge \forall i=1,\cdots ,N\\ \;1\;\forall j=2,\cdots ,N\wedge i=j-1\\ \;0\;\mathrm{otherwise} \end{array}\right..$$

Because the system in (16) is overdetermined, we propose solving it according to the LS criterion, which leads to

(18) $$b={\left(F^{T}F\right)}^{-1}F^{T}\Delta b\;.$$

Each value $b_{i}$ of vector b, for i=1,⋯,N, is stored in the corresponding anchor, and it is sent to the master anchor at the beginning of the localization phase. The master anchor uses the received values to compute the vector Δb, which is subtracted from (10) to compensate for the antenna delay bias.

## 4. Modified 1D IFA for Outlier Removal

Data acquired from real-world systems are usually affected by skewed or out-of-distribution samples. These samples, commonly called outliers, need to be properly handled to avoid a drastic reduction in localization performance. In this section, we describe a method to detect and filter such outliers from the ADS-TWR measurements. To tackle this problem, we applied a monodimensional (1-D) IFA [45], an unsupervised learning algorithm for anomaly detection. In the following, we first describe how the 1D IFA works, then we show how we adapted it to our specific settings.

Let us consider the problem of detecting outliers and separate them from a set $S=\{s_{1},\;\cdots \;,s_{N_{s}}\}$ of 1D data with cardinality $\left|S\right|=N_{s}$. The 1D IFA algorithm requires the definition of the following structures:

- **Isolation Tree (iTree)**: It is a binary tree, where each node has either zero or two child nodes. Nodes can be either external or internal depending on their position into the tree. An internal node is denoted as $\mathrm{intNode}(C_{L},C_{R},\alpha )$, where $C_{L}$ and $C_{R}$ are the left and right child nodes, respectively; and α is the split value that defines the separation between $C_{L}$ data and $C_{R}$ data. An external node is denoted as extNode(set,size), being thus defined on the set of data points belonging to the extNode and its cardinality.
- **Path length** is denoted as $P(s_{m})$; it measures the depth of the data point $s_{m}$ in the iTree. Outliers typically have shorter path lengths because they are more likely to be isolated.
- **Isolation forest**: It is a set composed by a fixed number $N_{F}$ of iTrees that are generated on the same set of data S.

**Figure 4:**
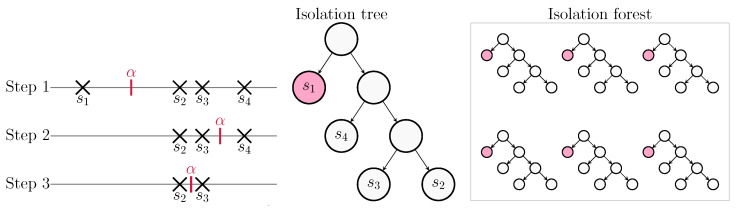
Isolation forest example with |D|=4.

A recursive procedure is used to generate an iTree. It splits the set S into $C_{L}$ and $C_{R}$ upon a random selection of the value α, and it is repeated until either |S|=1 or the depth of the node has reached a maximum length $L_{\mathrm{MAX}}$. The pseudo-code for the algorithm is shown in Algorithm 1. The isolation forest is then created by generating $N_{F}$ iTrees from the same input set S. The overall process for generating the Isolation forest is sketched in Figure 4.
**Algorithm 1:** iTree(S, *n*, $L_{\mathrm{MAX}}$)
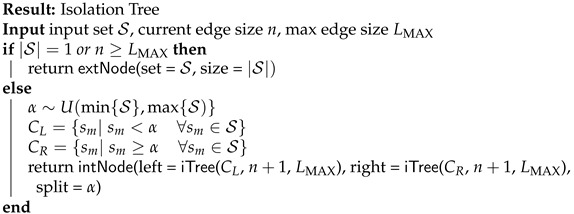


After the generation of the isolation forest, the score of each data point $s_{m}$ is computed as

(19) $$\mathrm{score}(s_{m},|S|)=2^{-\frac{E\{P\left(s_{m}\right)\}}{c(|S|)}}\;,$$

where $E\{P\left(s_{m}\right)\}$ is the expected value of the path length $P(s_{m})$; thus, it is the average length of each external node containing $s_{m}$, and c(x)=2C(x−1)−2(x−1)/x, with C(n)=ln(n)+0.5772156649 is the estimated value of the harmonic number. Intuitively, if  $\mathrm{score}(s_{m},|S|)$ is close to one, the corresponding data can be confidently marked as an anomaly. As a last step, it is necessary to define a threshold $\tau_{sh}$ such that a data point with $\mathrm{score}(s_{m},|S|)\geq \tau_{sh}$ is considered as potential outlier. The choice of $\tau_{sh}$ is important for the performance of the outlier detection. In [45], $\tau_{sh}=0.6$ was suggested. We tested different values of $\tau_{sh}$ and finally selected $\tau_{sh}=0.53$ as the the best one. We highlight that this value can be different if other scenarios are considered.

To adapt the IFA scheme to our case characterized by few UWB ranging measurements for each anchor pair, let us define the IFA input set as $S≜D_{ij}$, where $D_{ij}={\left\{{\hat{d}}_{ij}^{\left(m\right)}\right\}}_{m=1}^{M_{ij}}$ is the set of distance measurements between AP*i* and AP*j* defined in Section 3.1. The number of iTrees per isolation forest is $N_{F}=100$, while the maximum tree length $L_{\mathrm{MAX}}$ depends on the size of the input set $D_{ij}$, and it is calculated as $L_{\mathrm{MAX}}=\log_{2}\left(M_{ij}\right)$. The cardinality of the distance measurements set may impact on the performance for outlier removal. When $|D_{ij}|\leq 3$, it is unreliable to discriminate whether a data point is an outlier. Considering that the UWB system exchanges few ranging measurements for each anchor pair and many of them may be unsuccessful, we propose a modification of the original algorithm to tackle sets with very few data points.

In the modified IFA, we first calculate the cardinality $|D_{ij}|$ of the input set. If $|D_{ij}|>3$, we execute the normal IFA described before; otherwise, we measure the range of the set by making the difference between the maximum and the minimum value of the set $D_{ij}$ as

(20) $$\beta =\max\left\{D_{ij}\right\}-\min\left\{D_{ij}\right\}\;.$$

If β≤1 m, we left the set unchanged. Otherwise, the algorithm starts an iterative procedure by generating a uniform random number $\alpha ∼U(\min\{D_{ij}\},\max\left\{D_{ij}\right\})$ and separates $D_{ij}$ into two clusters, counting which data point is isolated. Because we have only three data points, the isolated one could be either $\max\{D_{ij}\}$ or $\min\{D_{ij}\}$. This separation is repeated for $N_{F}=100$ times, counting if the maximum value is isolated at each iteration, obtaining the number $N_{cm}$. We eliminate $\max\{D_{ij}\}$ if $N_{cm}\geq 90$ or min{S} if $(N_{F}-N_{cm})\geq 90$.

**Figure 5:**
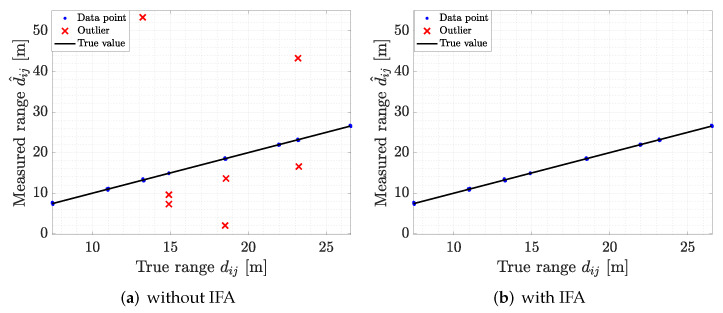
Outlier filtering by the proposed 1D IFA: (**a**) range measurements before outlier filtering and (**b**) after outlier filtering.

To illustrate the process of 1D IFA for outlier detection, we report an example of raw ADS-TWR measurements in Figure 5a, where outliers are marked with red crosses; in Figure 5b, we report the same ADS-TWR measurements after the outlier correction. Figure 5b highlights how the data points that are considered as outliers are removed by 1D IFA. Further analysis of the performance of the outlier detection algorithm is provided in Section 7.

## 5. Localization Methods

In this section, we detail the localization algorithms used for both anchors’ SL (Section 5.1) and tag localization (Section 5.2). Obtaining precise information on the AP locations is fundamental as they not only influence the tag position estimate but also the time-synchronization procedures. For this reason, despite being independent, the two localization algorithms are strongly paired, as the poor accuracy of anchors’ SL unavoidably prevents accurate tag positioning.

### 5.1. Anchors Self-Localization

We propose a SL procedure to estimate the AP positions. The automatization of this process avoids the need to manually measure each anchor-to-anchor distance to retrieve the overall anchor geometry, which is not only time consuming but also prone to human errors. SL is here performed using the ADS-TWR measurements estimated by the UWB system.

We propose an iterative algorithm, inspired by [26], to estimate the AP position $p_{i},\forall i\in \left\{1,\;\cdots \;,N\right\}$. We denote the estimate of $p_{i}$ as ${\hat{p}}_{i}$. The procedure assumes that the first two anchors are placed on the same line, and it exploits the estimated anchor-to-anchor distance averaged over all available measurements between AP*i* and AP*j*, i.e., ${\bar{d}}_{ij}=1/M_{ij}\sum_{m=1}^{M_{ij}}{\hat{d}}_{ij}^{\left(m\right)}$.

The proposed approach, summarized in the pseudo code of Algorithm 2, works as follows: At first, two anchors, out of the total *N* available, are assumed to be positioned at ${\hat{p}}_{1}={\left[0\;\;0\right]}^{T}$ and ${\hat{p}}_{2}={\left[{\bar{d}}_{1,2}\;\;0\right]}^{T}$ (note that one could choose to deploy the two initial APs along the orthogonal axis, i.e., ${\hat{p}}_{1}={\left[0\;\;0\right]}^{T}$ and ${\bar{p}}_{2}={\left[0\;\;{\bar{d}}_{1,2}\right]}^{T}$, obtaining an equivalently valid final solution). We then iteratively search for the position of the remaining anchors by applying the GN algorithm. For the *i*th AP, with i=3,⋯,N, we initialize its estimate ${\hat{p}}_{i}$ in a random location within a square area of dimension $\max\left(\hat{D}\right)\times \max\left(\hat{D}\right)$ and origin (0,0), with  $\bar{D}=\left\{{\bar{d}}_{ij}:1\leq i\leq N,1\leq j\leq N,i\neq j\right\}$. We then iteratively update the estimate ${\hat{p}}_{i}$ as

(21) $${\hat{p}}_{i}={\hat{p}}_{i}+{\left(G^{T}G\right)}^{-1}G^{T}\left(r-g\left({\hat{p}}_{i}\right)\right)\;,$$

where $r={\left[{\bar{d}}_{i,1}\cdots {\bar{d}}_{i,i-1}\right]}^{T}$ collects the ADS-TWR measurements of preceding APs (i.e., up to index i−1), $g\left({\hat{p}}_{i}\right)=[∥{\hat{p}}_{i}-{\hat{p}}_{1}∥\cdots ∥{\hat{p}}_{i}-{\hat{p}}_{i-1}{∥]}^{T}$ and $G={\left[\partial g\left({\hat{p}}_{i}\right)/\partial {\hat{p}}_{1}\cdots \partial g\left({\hat{p}}_{i}\right)/\partial {\hat{p}}_{i-1}\right]}^{T}$. This procedure is repeated until convergence, i.e., when the improvement $\left|\Delta p|=\right|{\left(G^{T}G\right)}^{-1}G^{T}\left(r-g\left({\hat{p}}_{i}\right)\right)|$ over the previous iteration is below a threshold δ or a maximum number of iterations $I_{max}$ is reached. Once a first position estimate is obtained for all anchors, the algorithm is run a second time to improve the localization accuracy. Now, each anchor is estimated taking into account all other anchors rather than considering only the preceding ones. More specifically, if we consider anchor AP*i* to be currently positioned, in the first iteration, it is updated considering the anchors up to APi−1, while now it is estimated considering all other anchors except AP*i*. This improves the performances of the SL procedure, especially for the first anchors, i.e., for i≪N. In the second iteration, the updated AP position estimate is still performed using (21), but now with variables $r={\left[{\bar{d}}_{i,1}\cdots {\bar{d}}_{i,i-1}\;\;\;{\bar{d}}_{i,i+1}\cdots {\bar{d}}_{i,N}\right]}^{T}$, $g\left({\hat{p}}_{i}\right)=[∥{\hat{p}}_{i}-{\hat{p}}_{1}∥\cdots ∥{\hat{p}}_{i}-{\hat{p}}_{i-1}∥\;\;\;∥{\hat{p}}_{i}-{\hat{p}}_{i+1}∥\cdots ∥{\hat{p}}_{i}-{\hat{p}}_{N}{∥]}^{T}$ and $G={\left[\partial g\left({\hat{p}}_{i}\right)/\partial {\hat{p}}_{1}\cdots \partial g\left({\hat{p}}_{i}\right)/\partial {\hat{p}}_{i-1}\;\;\;\partial g\left({\hat{p}}_{i}\right)/\partial {\hat{p}}_{i+1}\cdots \partial g\left({\hat{p}}_{i}\right)/\partial {\hat{p}}_{N}\right]}^{T}$. A metric is introduced to evaluate how close the distances, extracted from the newly positioned anchors, are to the available TOA measurements. Formally, it is computed as

(22) $$\epsilon =\sum_{i=1}^{N}\sum_{j\neq i}^{N}\frac{(∥{\hat{p}}_{i}-{\hat{p}}_{j}{∥-{\bar{d}}_{ij})}^{2}\;}{N(N-1)}\;.$$

The final AP positions ${\hat{p}}_{i},\forall i\in \left\{3,\;\cdots \;,N\right\}$ are obtained once $\epsilon <\epsilon_{\min}$ or when the maximum number of iterations $I_{max}$ is reached. It should be noted that (22) assesses only how close or far the relative distances of the anchors are with respect to the TOAs. Thus, obtaining precise TOA measurements among APs is of fundamental importance. This aspect is further analyzed in Section 7 to show how the performance varies when directly estimating the anchors’ positions from uncorrected TOAs, after applying the ADS-TWR antenna delay procedure and after removing any outlier from the ranging measurements.
**Algorithm 2:**Autolocalization($\bar{D},\epsilon_{min},I_{max},\delta$)
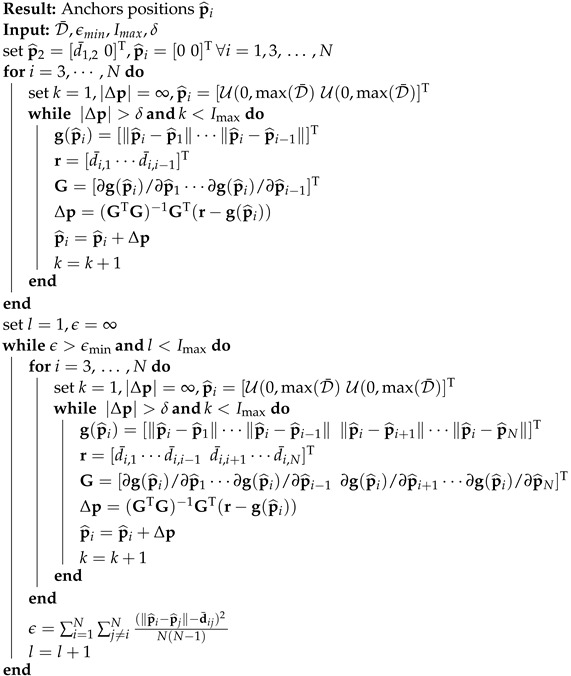


### 5.2. Tag Localization

Tag localization follows a LS approach, with GN and LM implementations. Given the tag position $u\in R^{3}$, its estimate $\hat{u}$ is computed by minimizing the sum of squared residuals as

(23) $$\hat{u}=\arg\min_{u}∥\rho -h(u){∥}^{2}\;,$$

and the solution of (23) can be obtained iteratively (an iteration is indicated with subscript *k*) through both GN and LM algorithms as described in the following. First, let us suppose that a previous solution $u_{k-1}$ is available, then the position using the GN algorithm is updated as

(24) $${\hat{u}}_{k}=u_{k-1}+{\left(H{\left(u_{k-1}\right)}^{T}H\left(u_{k-1}\right)\right)}^{-1}H{\left(u_{k-1}\right)}^{T}\left(\rho -h\left(u_{k-1}\right)\right)\;,$$

where

(25) $$H\left(u_{k-1}\right)={\left[\partial h_{1}\left(u\right)/\partial u\cdots \partial h_{N}\left(u\right)/\partial u\right]}^{T}{|}_{u=u_{k-1}}.$$

The algorithm starts from a random initial solution and stops after reaching a maximum number of iterations $I_{\mathrm{MAX},\mathrm{GN}}$ or when the residual $∥{\hat{u}}_{k-1}-u_{k-1}∥$ is lower than a certain threshold.

The GN algorithm, however, has poor convergence properties, especially when the Hessian matrix $H{\left(u_{k-1}\right)}^{T}H\left(u_{k-1}\right)$ is close to singular or ill-conditioned. In these cases, the update is not defined, and the algorithm diverges from the optimal solution. This problem is overcome by the use of LM algorithm [47], which amounts to adding a damping term $\lambda I_{3}$ to the Hessian matrix, which guarantees the existence of its inverse and allows us to rewrite (24) as

(26) $${\hat{u}}_{k}=u_{k-1}+\Delta u=u_{k-1}+{\left(H{\left(u_{k-1}\right)}^{T}H\left(u_{k-1}\right)+\lambda I_{3}\right)}^{-1}H{\left(u_{k-1}\right)}^{T}\left(\rho -h\left(u_{k-1}\right)\right)\;.$$

The damping parameter influences how the algorithm updates the location estimate. Specifically, when λ is small, the algorithm is roughly identical to the GN; when λ is large, it behaves as the gradient descent algorithm [60]. The LM method tries to combine the strengths of both approaches: it has a faster convergence with respect to the gradient descent algorithm, while not suffering from the divergence problems of the GN. The damping parameter is adapted at each iteration using an auxiliary factor ν, so as to steer convergence. More specifically, the algorithm evaluates the update step Δu in (26) and computes

(27) $$\gamma =\frac{∥\rho -h\left(u_{k-1}\right){∥}^{2}-{∥\rho -h\left(u_{k-1}+\Delta u\right)∥}^{2}}{0.5\;\Delta u^{T}\left(\lambda_{k-1}\Delta u+H{\left(u_{k-1}\right)}^{T}\left(\rho -h\left(u_{k-1}\right)\right)\right)}\;,$$

where $\lambda_{k-1}$ is the damping parameter at the previous iteration. Depending on the value of γ the position is updated. In particular, the step Δu is considered valid if γ>0, because $∥\rho -h\left(u_{k-1}\right){∥}^{2}$ decreases compared with the last iteration; otherwise, the position is not updated. Thus, for strictly positive values of γ, the new position is computed from (26), and the damping factor is updated as

(28) $$\lambda_{k}=\lambda_{k-1}\max\left\{\frac{1}{3},1-{\left(2\gamma -1\right)}^{3}\right\}.$$

On the other hand, if γ<0, ${\hat{u}}_{n}=u_{k-1}$, $\lambda_{=}\lambda_{k-1}\;\nu_{k}$ and $\nu =2\;\nu_{k-1}$, with $\nu_{k-1}$ being the factor at the previous iteration. At the first iteration, $\lambda_{0}=\tau \max\{\mathrm{diag}\left(H\left(u_{0}\right)\right\}$, with τ=5 and $\nu_{0}=2$ [47]. The LM procedure starts with a random guess and iteratively updates the estimated position until a prefixed number of iterations $I_{\mathrm{MAX},\mathrm{LM}}$ has been reached or the step |Δu| is below a threshold. For sport applications, the tag localization is usually performed in 2D; however, we performed the tag localization also considering the *z* axis. The reason for the estimation of the *z* coordinate is that anchors and tags do not lie on the same plane, so the height differences between a tag and an anchor can introduce a bias in the position estimation if it is not considered. This effect is particularly evident when a tag is very close to the anchor position. If we consider the *z* axis, this problem can be avoided.

## 6. Experimental Setup and Performance Metrics

In this section, we describe the technical features of the UWB devices used in the experiments, followed by the definition of performance metrics.

**Figure 6:**
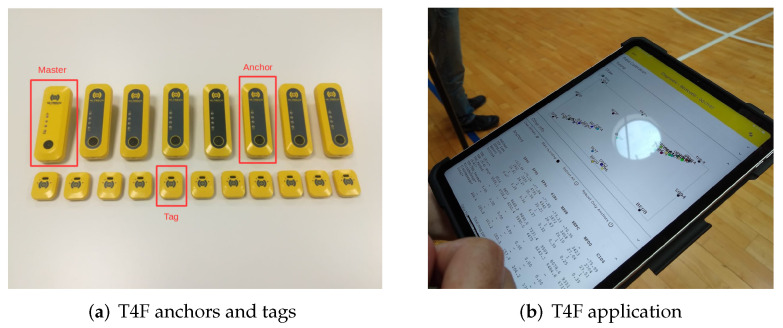
(**a**) T4F UWB modules (anchors and tags). (**b**) T4F application running on a tablet.

We used a proprietary UWB system composed of anchors and tags, as depicted in Figure 6a. The UWB system was engineered by Tracking4Fun S.r.l., Florence, Italy. Anchors are equipped with the Decawave DWM1000 module, which provides ranging estimation capabilities. The DWM1000 module is an integrated circuit composed of a DW1000 wireless transceiver, an antenna, and a printed circuit board. The radio transceiver is designed as a single CMOS chip, and it is compliant with the IEEE 802.15.4-2011 UWB standard [59]. It supports six channel bands ranging from 3.5 GHz to 6 GHz with a bandwidth of either 499.2 or 1331.2 MHz, and providing three data rates: 110 kb/s, 850 kb/s, and 6.8 Mb/s. The module could be configured to support Two-Way Ranging (TWR) procedures via TOF estimation or TDOA with a nominal coverage of 60 m. APs were configured to use channel 4, with 1331.2 MHz bandwidth, a central frequency of 3993.3 Mhz, and a bit rate of 850 kb/s. On the other hand, tags were equipped with a Decawave DWM1001 module, also based on the DW1000 wireless transceiver. In contrast to DWM1000, DWM1001 supports only channel 5 with a 6489.2 MHz central frequency, 499.2 MHz bandwidth, and bit rate of 6.8 Mb/s. The module integrates also a Nordic microprocessor nRF52832 Bluetooth antenna and a STMicroelectronics LIS2DH12TR triaxial accelerometer. The achievable TDOA refresh rate and, consequently, the location rate depend on the number of active tags: the channel access used a Time Division Multiple Access (TDMA) scheme limiting the location rate changes from 0.01667 to 10 Hz, corresponding to a number of active tags of 9000 and 15, respectively. As for the anchors, the module could be configured to support TWR procedures via TOF estimation or TDOA estimation. Additionally, tags had a nominal coverage of 60 m.

**Figure 7:**
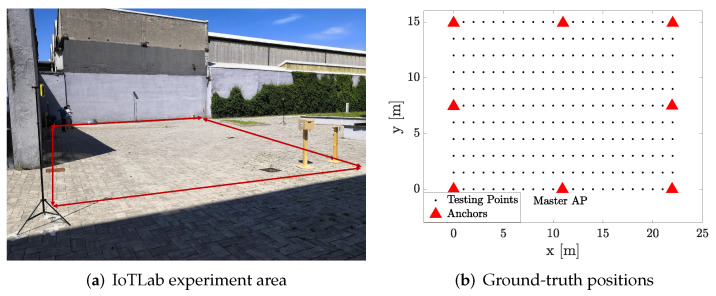
Experimental area: (**a**) picture of the area; (**b**) ground-truth positions of tags and anchors.

An experimental campaign was carried out at the IoTLab of Politecnico di Milano to assess the performance of the UWB system. Figure 7a depicts the selected area for the experiments. Eight anchors were deployed at a height of 1.9 m, and their relative distances were measured with a laser meter to ensure highly accurate ground truth information. The ground-truth positions of tags were selected according to the grid reported in Figure 7b, which had a horizontal spacing of 1 m and a vertical one of 1.5 m, for an overall number of 23×11 evaluation points.

The UWB localization experiment was conducted as follows. For each vertical line in the grid, we deployed 2 tags at each position and recorded raw UWB data for 45 s, resulting in 220 TDOA measurements for each tag, on average. The 22 tags were divided into two groups: 12 tags (first group) were attached to cardboard boxes at a height of 1.3 m, while the remaining 10 were worn by people. During the registration, people changed their orientation by 90 degrees counterclockwise every 15 s to avoid any biases due to the human posture, while cardboard boxes did not rotate. Once the registration for a single vertical line was terminated, we moved to the adjacent line and started a new recording. Each simulation was independent from the others and, at the end, all the $N_{r}=23$ registrations were aggregated for a more robust statistical analysis.

We evaluated the performance of the proposed algorithms with the following metrics. Regarding outlier detection, we assessed it as a binary classification problem, thus measuring the algorithm capability to successfully identify an outlier or a failure. Considering the error $e_{d}={\hat{d}}_{ij}-d_{ij}$ of a ranging measurement computed as the difference between the real distance and the measurement between AP*i* and AP*j*, we discriminated the outliers by computing the lower and upper adjacent defined as

(29) $$q_{l}=\mathrm{LQ}-1.5\;\left(\mathrm{UQ}-\mathrm{LQ}\right)$$

(30) $$q_{u}=\mathrm{UQ}+1.5\;\left(\mathrm{UQ}-\mathrm{LQ}\right)$$

where UQ and UQ are the first and third quartile, respectively, evaluated as

(31) $$\mathrm{Pr}(e_{d}\leq \mathrm{LQ})=0.25$$

(32) $$\mathrm{Pr}(e_{d}\leq \mathrm{UQ})=0.75\;.$$

We then marked a measurement as an outlier if the error was either smaller than the lower adjacent or higher than the upper adjacent. Framing the outlier detection as a classification problem, we used the widely adopted metrics of sensitivity, specificity, and accuracy to evaluate the performance. The sensitivity $S_{v}$ measures the ratio between the correctly detected positive values (true positive (TP)) and the total number of positive values, and it is computed as

(33) $$S_{v}=\frac{\mathrm{TP}}{\mathrm{TP}+\mathrm{FN}}\;,$$

where FN is false negative. The specificity $S_{p}$ measures the ratio of the number of correctly detected negative values (true negative (TN)) and the total number of negative values, and it is computed as

(34) $$S_{p}=\frac{\mathrm{TN}}{\mathrm{TN}+\mathrm{FP}}\;,$$

where FP is false positive. The accuracy $A_{cc}$ measures the number of correctly classified measurements and is computed as

(35) $$A_{cc}=\frac{\mathrm{TN}+\mathrm{TP}}{\mathrm{TN}+\mathrm{TP}+\mathrm{FN}+\mathrm{FP}}\;.$$

The evaluation of SL considers the 2D AP position error. Given the true AP position $p_{i}$ and its estimate ${\hat{p}}_{i}$, the position error $e_{p,i}$ is

(36) $$e_{p,i}=\sqrt{{\left({\hat{p}}_{i,x}-p_{i,x}\right)}^{2}+{\left({\hat{p}}_{i,y}-p_{i,y}\right)}^{2}}\;.$$

Similarly, tag localization was evaluated with the 2D positioning error $e_{u}$, computed as

(37) $$e_{u}=\sqrt{{\left({\hat{u}}_{x}-u_{x}\right)}^{2}+{\left({\hat{u}}_{y}-u_{y}\right)}^{2}}\;.$$

Average performance was computed by aggregating data from $N_{r}$ registrations in terms of Root Mean Square Error (RMSE), Circular Error Probable (CEP) at 95% of confidence (CEP 95), and mean error.

## 7. Results and Discussion

**Figure 8:**
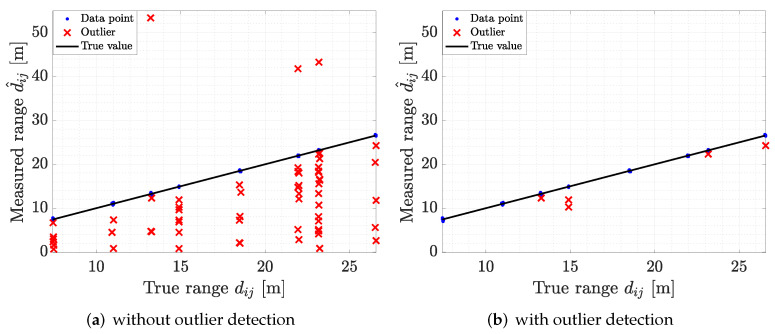
Performance of outlier detection algorithm for ranging measurements: (**a**) without outlier detection; (**b**) with outlier detection.

In this section, we discuss the performance assessment and validation of all the proposed methods, for both SL and tag localization. As a first analysis, we evaluated the performance of the proposed outlier detection algorithm employed for filtering out anomalies in ranging data. We report in Figure 8a the raw ADS-TWR measurements gathered during the experimental campaign, indicating the outliers with red crosses. We found that a high number of outliers needed to be filtered to limit the impact on SL. The application of the proposed 1D IFA method allowed us to significantly reduce the number of anomalies in the ranging data, as highlighted by the result in Figure 8b.

The validation analysis had a sensitivity of $S_{v}=0.9231$, a specificity of $S_{p}=0.9995$, and an accuracy of $A_{cc}=0.9982$. The values of sensitivity and specificity indicated that the proposed method was able to correctly discriminate the outliers from data, and detecting almost all of them. The accuracy provides a more general view on the designed method performance, showing that the algorithm provided correct predictions for over 99% of the data. Overall, the detection algorithm was able to eliminate most of outliers without removing correct data, allowing for a precise estimation of the TOA measurements and, consequently, leading to better anchor and tag localization.

**Figure 9:**
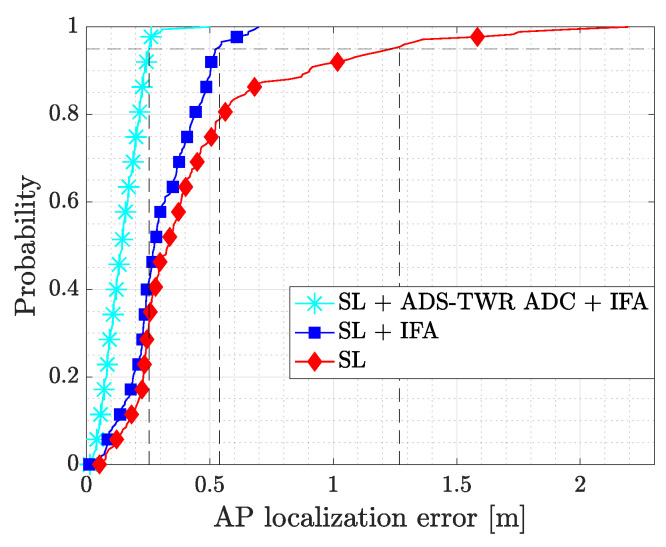
CDF of the anchors’ SL position error considering different levels of correction. Dashed lines highlight the CEP 95 values.

In the analysis of the SL algorithms, we considered the following three cases: a first one where the antenna delay bias was not corrected and the outlier detection algorithm was absent (SL); a second one where the antenna delay bias was not corrected but the outlier detection algorithm was applied (SL + IFA); a third one where both corrections were applied (SL + ADS-TWR ADC + IFA). This allowed us to separately analyze the performance improvement introduced by the calibration step and by the anomaly detection. For all considered cases, we computed the anchor position error $e_{p}$ and the associated RMSE, CEP, and mean error. Table 1 reports the achieved value for the three cases, while Figure 9 focuses on the Cumulative Distribution Function (CDF) of $e_{p}$. Comparing the results, the implementation of 1D IFA for outlier detection alone considerably improved the localization performances compared with the plain SL algorithm with no corrections applied. The RMSE reduced by approximately 30 cm, the mean error by 16 cm, and the CEP 95 by 80 cm. This definitely proved the importance of the anomaly detection process. An additional improvement was observed when the outlier detection algorithm was combined with the ADC: the RMSE, mean error, and CEP 95 were further reduced by 17 cm, 15 cm and 30 cm, respectively. The combination of the ADC and IFA outlier removal algorithm was able to provide highly accurate anchors positions, which are necessary for implementing a precise positioning system.

After analyzing the performance of SL, we focused on tag localization. The analysis aimed to highlight the impact of applying the ADC, as well as the effect of the SL algorithm on the position accuracy, and the performance improvement obtained by applying the ADS-TWR ADC.

**Figure 10:**
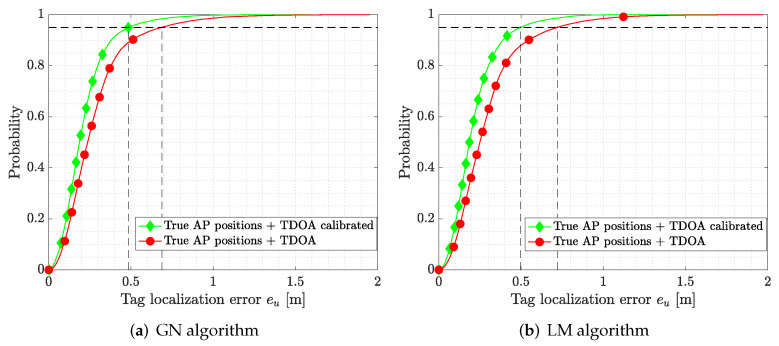
CDF of the tag localization error $e_{u}$ for GM and LM algorithms with and without TDOA bias correction. Dashed lines indicate the CEP 95 values.

We first considered the TDOA bias correction on both GN and LM algorithms; the effect of error corrections on tag localization is shown in Figure 10a for the GN algorithm and in Figure 10b for the LM one, where we provide the CDF of the tag localization error $e_{u}$. Comparing the results, it followed that by correcting the TDOA bias, it was possible to reduce the tag localization error and increase the accuracy of the system. In particular, the CEP 95 reduced from 68 cm to 49 cm for the GN algorithm, and from 72 cm to 50 cm for the LM one. All the metric values are reported in Table 2.

To show the impact of the SL algorithm on the localization accuracy, we assessed the performance of the LS solution with and without the ADS-TWR delay correction. Furthermore, the effect of ADS-TWR outliers on localization was addressed. We used a baseline approach to benchmark the performance, where the true positions of the APs were used. We also considered the TDOA bias correction on these data.

**Figure 11:**
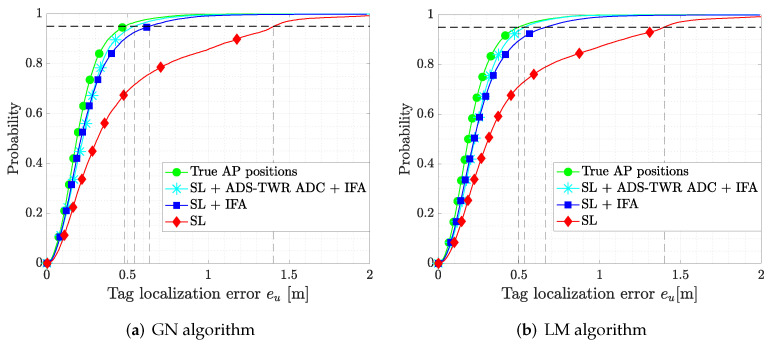
CDF of the localization error $e_{u}$ for GN and LM algorithms considering different corrections.

In Figure 11a,b, we report the CDF of the tag location error $e_{u}$ for the GN algorithm and the LM algorithm, respectively, showing the improvement in the proposed corrections with respect to the baseline case (i.e., SL). The outlier detection (SL + IFA) improved the system performance, and further enhancements are obtained when the ADS-TWR ADC is used (SL + ADS-TWR ADC + IFA). The achievable tag localization error of both algorithms with bias compensation are very close to the case of using the true AP positions, highlighting again the importance of the calibration step. The metrics of all algorithms are reported in Table 3.

**Figure 12:**
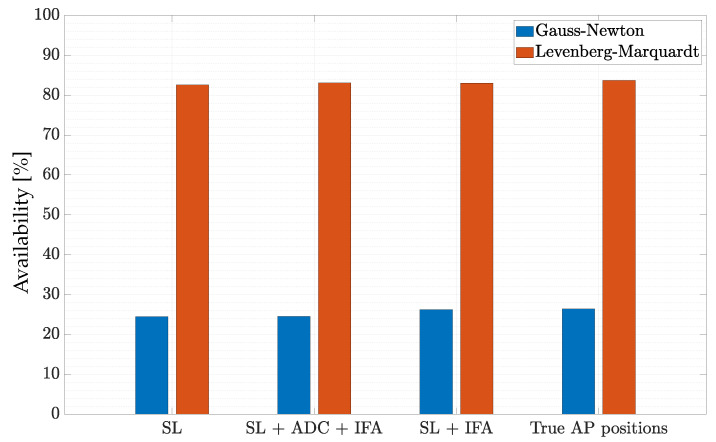
Availability of the GN and LM algorithms.

As a last analysis, we compared the GN and LM algorithms in terms of availability in providing positioning estimates. The availability is expressed as the number of times the LS algorithm (either GN or LM) converged with respect to the total number of runs of the algorithm. For this experiment, we set $I_{\mathrm{MAX},\mathrm{GN}}=I_{\mathrm{MAX},\mathrm{LM}}=100$. We depict the bar plot of the availability for both GN and LM in Figure 12, considering all the correction strategies for antenna delay and outliers. The availability for the GN algorithm was lower than 30% in all conditions, indicating that more than the 70% of the time the algorithm did not converge. This was due to the fact that the Hessian matrix $H{\left(u\right)}^{T}H\left(u\right)$ in (24) was singular most of the time. The problem was easily solved using LM algorithm, which showed an availability that was always higher than 80%.

## 8. Conclusions and Future Studies

In this paper, we presented a localization system based on UWB technology, mainly targeting sport applications or other use cases characterized by temporary installations. We developed several techniques aimed at increasing the positioning performance of the system, both for anchors’ self-localization and real-time tag positioning. Specifically, we developed a GN method to compensate for the antenna delay at the anchors, an improved version of the isolation forest algorithm targeting the removal of the outliers from low-dimensional ADS-TWR sets, as well as a novel self-localization algorithm for accurate anchors’ position calibration.

The proposed techniques were validated considering an outdoor experimental campaign at IoTLab, Politecnico di Milano, where raw UWB data were collected. The results showed that the combination of the aforementioned techniques substantially improved the positioning accuracy compared with a conventional system that did not employ any ADC and outlier removal operation. By sequentially integrating ADC and outlier removal into the localization framework, the error reduced from 1.3 m to 25 cm for self-localization positioning, whereas from 1.4 m to 55 cm for tag positioning, indicating that a proper handling of the outliers and the compensation of the antenna delay are required to obtain high-accuracy location estimates. We managed to achieve localization performance comparable to that of exactly knowing the anchors positions (i.e., when the deployment is fixed), minimizing the errors introduced by the self-localization.

In future studies, we will consider the use of Bayesian filtering for further performance enhancement of tag localization by leveraging well-calibrated motion models for each specific use case. Moreover, different use cases and experimental campaigns may be needed to fully characterize the robustness of the localizing system considering challenging indoor scenarios characterized by harsh propagation conditions as well as unfavorable AP geometries.

## Figures and Tables

**Table 1:** Performance metrics of SL with/without outlier and ADS-TWR antenna delay corrections.

	RMSE (m)	CEP 95 (m)	Mean Error (m)
SL	0.6154	1.3068	0.4708
SL + IFA	0.3365	0.5223	0.3056
SL + ADS-TWR ADC + IFA	0.1626	0.2482	0.1463

**Table 2:** Performance metrics of tag localization for GN and LM algorithms with real AP positions and with/without TDOA antenna delay correction.

	RMSE (m)	CEP 95 (m)	Mean Error (m)
GN with true AP positions	0.3509	0.6887	0.2817
GN with true AP position + TDOA ADC	0.2817	0.4875	0.2181
LM with true AP positions	0.3654	0.7208	0.2959
LM with true AP positions + TDOA ADC	0.2617	0.4965	0.2176

**Table 3:** Comparison of GN and LM algorithms for tag localization with/without outlier and antenna delay corrections.

	RMSE (m)	CEP 95 (m)	Mean Error (m)
GN with true AP positions	0.2817	0.4875	0.2181
GN with SL	0.6316	1.4089	0.4842
GN with SL + IFA	0.3447	0.6381	0.2660
GN with SL + ADS-TWR ADC + IFA	0.3072	0.5584	0.2487
LM with true AP positions	0.2617	0.4965	0.2176
LM with SL	0.6606	1.3991	0.4635
LM with SL + IFA	0.3447	0.6887	0.2770
LM with SL + ADS-TWR ADC + IFA	0.2993	0.5335	0.2494

## Data Availability

Not applicable.

## Abbreviations

The following abbreviations are used in this manuscript:

**Abbreviation**	**Definition**
ADC	Antenna Delay Calibration
ADS-TWR	Asymmetric Double-Sided Two-Way Ranging
AOA	Angle of Arrival
AP	Access Point
CEP	Circular Error Probable
CDF	Cumulative Distribution Function
GN	Gauss–Newton
GNSS	Global Navigation Satellite System
IFA	Isolation Forest Algorithm
IoT	Internet of Things
LM	Levenberg–Marquardt
LS	Least Square
PSO	Particle Swarm Optimization
RMSE	Root Mean Square Error
RSS	Received Signal Strength
RTT	Round Trip Time
SL	Self-Localization
TDMA	Time Division Multiple Access
TDOA	Time Difference of Arrival
TOA	Time of Arrival
TOF	Time of Flight
TWR	Two-Way Ranging
UWB	Ultrawide-Band
WSN	Wireless Sensor Network
